# Domestic Horse Bite: An Unusual Etiology of Crush Injury of the Fourth Finger—How to Manage?

**DOI:** 10.1155/2019/2156269

**Published:** 2019-01-30

**Authors:** Naoufal Elghoul, Youssef Jalal, Ayoub Bouya, Ali Zine, Abdeloihab Jaafar

**Affiliations:** Orthopedic Surgery and Traumatology, Department of Orthopedic Surgery and Traumatology, Military Hospital Mohammed V (HMIMV), BP 10100 Rabat, Morocco

## Abstract

Almost 2% of all emergency admissions involve an animal bite. While horses bite humans very rarely, their bites are mostly associated with fatalities. Herein, we report the case of a 23-year old bitten by a domestic horse causing a crush injury to his fourth finger with fracture dislocation of the proximal interphalangeal joint. The patient benefited upon arrival at the emergency department from copious irrigation with saline serum, tetanus toxoid, postexposure rabies vaccination, and prophylactic antibiotic therapy. In the operating room, surgical exploration found the ulnar digital pedicle sectioned, the flexor and extensor tendons sectioned and shredded, and the skin shredded. An excisional debridement of devitalized tissue with copious irrigation was performed, and the finger regularized at the level of traumatic amputation with tendon striping followed by coverage of the bone by the radial digital flap with careful clinical and biological monitoring after the surgery. At the last follow-up, the patient revealed no sign of infection, and he returned to his usual activities and has been discharged from care. This wound management, based on a careful examination, a meticulous debridement, and an efficient cleaning with early and targeted antibiotic therapy, might promote good results and avoid dangerous complications.

## 1. Introduction

Animal bites can cause complications ranging from slight injuries to serious infections. Almost 2% of all emergency admissions involve an animal bite [1].

Dog bites are the most frequent (80%–90%) of animal bites, followed by cat and then human bites, with donkeys and horses rarely biting humans [2]. Although rare, horses are the animal that is most commonly involved in bite-related fatalities [3].

Herein, we report an extremely rare case that illustrates the severity of horse bites, and how they can be best managed.

## 2. Case Presentation

**Figure 1:**
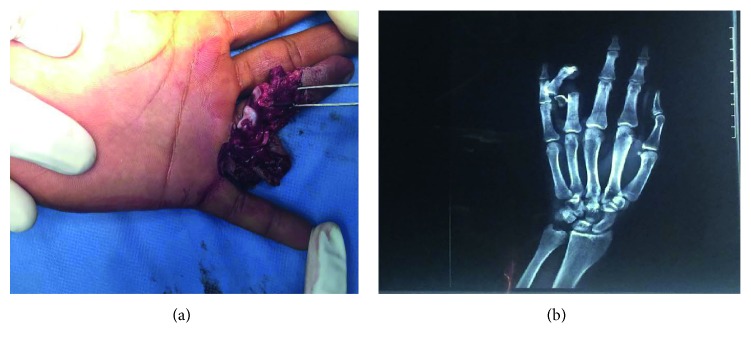
(a) Clinical aspect of the crush injury of the fourth finger. (b) X-rays showed a fracture dislocation of the proximal interphalangeal joint with third fragment.

A 23-year-old male was feeding his horse, and while stroking the horse's hair, the animal chewed the fourth finger of his left hand causing violent pain and total functional impotence of the finger. Both the patient and the horse were up to date on their required vaccinations at the time of the incident. The patient was transferred to an emergency department and was admitted six hours after the incident. He was conscious, in good general condition, and apyretic. An examination revealed a crush injury of the fourth finger with tendons and bone exposed (Figure 1(a)).

Copious irrigation with normal saline (2 liters) at the injury site was performed along with injection of 0.5 ml tetanus toxoid and 500 IU of human tetanus immunoglobulin. Postexposure rabies prophylaxis (rabies immune globulin human 20 IU/kg) with the first-dose rabies vaccine was injected into the depth of the wound as well as around the wound. The remaining rabies immune globulin was injected into the deltoid muscle. The patient was also treated with prophylactic antibiotic therapy with intravenous amoxicillin-clavulanate, gentamicin, and metronidazole.

**Figure 2:**
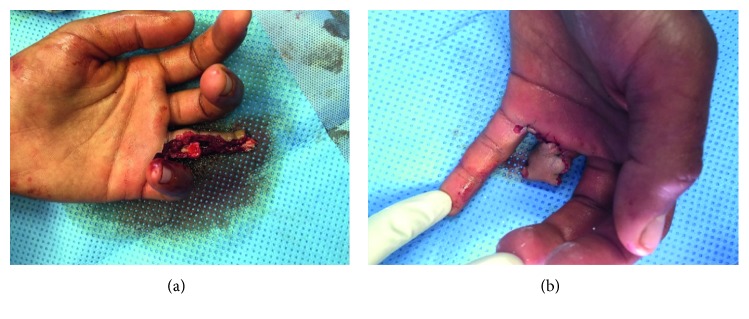
(a) Clinical aspect after debridement and regularization at the level of traumatic amputation. (b) Coverage of the bone by the radial flap using separated stitches.

After this initial treatment, radiography revealed a fracture dislocation of the proximal interphalangeal joint of the fourth finger with a third fragment (Figure 1(b)), prompting the patient to undergo surgery. Surgical exploration under locoregional anesthesia found that the ulnar digital pedicle was sectioned and thrombosed, the radial digital pedicle was intact, the flexor and extensor tendons were sectioned and shredded, and the skin was irreparably shredded (Figure 2(a)).

Surgical procedures included removal of foreign bodies and excisional debridement of devitalized tissue, collection of bacteriological samples, copious irrigation with saline serum (3 liters), tendon striping, and finger amputation with coverage of the bone by the radial digital flap using separate stitches (Figure 2(b)). The surgery was followed by careful clinical and biological monitoring.

A clinical assessment of the patient 1  day postoperatively showed that he was apyretic with no necrosis of the flap and no purulent discharge. A neurovascular examination was normal. Biological findings at this time showed a C‐reactive protein level of 30 mg/dL and a white blood cell count of 11000/*μ*L.

Three days postoperatively, bacteriological samples found an evidence of *Pasteurella* species and *Staphylococcus* sensitive to amoxicillin + clavulanic acid with C-reactive protein levels of less than 10 mg/dL and a white blood cell count of 7000/*μ*L. At this time, the patient received the second dose of rabies vaccine.

**Figure 3:**
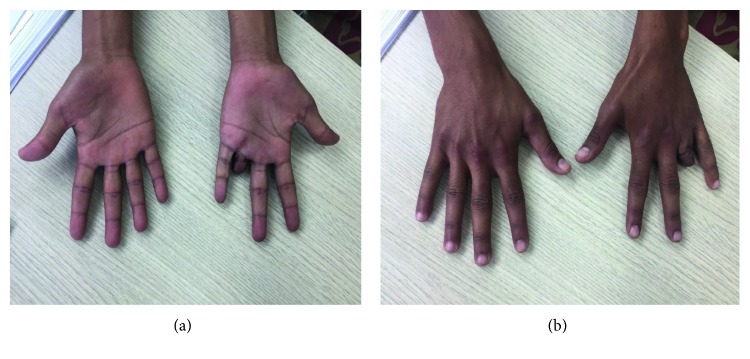
(a, b) Clinical aspect at a follow-up of three months.

One week postoperatively, the patient was discharged with a prescription for a course of 10 days amoxicillin-clavulanic acid treatment to be reviewed weekly. At a three-month follow-up (Figures 3(a) and 3(b)), the patient showed no sign of infection; he returned to his usual activities and was discharged from care.

## 3. Discussion

Although considered uncommon, equines may occasionally inflict bites on humans. Of all reported injuries involving horses, approximately 3% to 4.5% are related to bites [4]. Because an equine can exert a great deal of force in closing its jaws, the severity of injuries may range from minor superficial contusions, abrasions, or lacerations to severe crushing or tearing injuries with damage to deeper structures [5]. Horse-bite infections are typically polymicrobial with a mix of aerobic and anaerobic species. The *Actinobacillus* species are Gram-negative coccobacilli that are part of the normal oral flora of horses and common pathogens in infected bites. Other common pathogens include *Staphylococcus* species, *Streptococcus* species, and *Pasteurella* species, as well as *Fusobacterium* and *Bacteroides* species [6, 7]. In our case of crush injury of the fourth finger, we found an evidence of *Pasteurella* species and *Staphylococcus* in bacteriological samples.

The patients at the highest risk of infection are those with immune dysfunction of any cause (AIDS, hepatopathy, asplenism, cancer, neutropenia, diabetes, and treatment with corticosteroids or immunosuppressants) [8]. However, persons without any predisposing factors can also suffer from severe or lethal infections in bite wounds [9] which may include our patient.

When the horse bite occurs, it is important to determine the circumstances of bite, health, and vaccination status of the animal ( if known) and whether the animal is available for observation, also to determine the patient's vaccination status and the presence of risk factors for infection [10]. In our case, the patient was bitten while feeding his horse and both of them were up to date on their required vaccinations at the time of the incident. If there is a likelihood of damage to underlying bones and joints, such as with the hand, the radiographs should be obtained, and also the laboratory tests should be realized (e.g., complete blood count, CRP/PCT, and blood cultures) [11].

The rabies postexposure prophylaxis is indicated for all patients who are possibly exposed to a rabid animal; it is generally not required for patients bitten by animal who do not demonstrate signs of rabies for at least 10 days of monitoring [12]. Our patient received a rabies postexposure prophylaxis for more security because we had no idea about the observation of the horse at the time of the incident.

The wound management consists of cleaning, irrigation, exploration for foreign body or injury to deeper structures, and debridement [10]. For irrigation, it is usually made with normal saline and a few authors recommend antiseptic solutions [13]. As it may lead to the uncontrolled spread of bacteria into deeper tissue layers, irrigation under pressure is inadvisable [14]. For surgical debridement of the limb, it is recommended to be extensive [15]. For wound closures on the limbs, the existing recommendations are still inconsistent [16]. Our patient has benefited initially from the first cleaning by a normal saline and then acheminated to the operative room for an extensive debridement with copious irrigation and we elected to close the wound as the patient was young, immunocompetent and was presented early. A follow-up of 24 to 48 hours is indicated to review wound healing and monitor for signs of infection [10].

The use of antibiotic prophylaxis has not been shown to reduce the rate of infection, except in cases of patients at high risk of infection or in the case of bite wounds on hands [17]. Our patient presented a bite wound on hand that is why he received an early antibioprophylaxis. In contrast, antibiotics need not to be given if the patient presents 24 hours or more after the bite, and there are no clinical signs of infection [18]. Amoxicillin-clavulanic acid is appropriate for bites from most species and involving most anatomic locations. Alternatives include clindamycin, doxycycline, fluoroquinolones (e.g., ciprofloxacin and moxifloxacin), or cefuroxime [12]. When bite infection is declared, the duration of targeted antibiotic treatment recommended is generally 7 to 14 days until the infection has resolved [11].

At last, what had promoted good results and avoided dangerous complication in our case was the combination of careful examination, meticulous debridement, and efficient cleaning with early and targeted antibiotic therapy followed by close monitoring.

## 4. Conclusion

Because of the unique anatomy of the hand and the bacteriology of horse's oral flora, bite injuries can lead to severe infection and functional impairment. Early and rigorous management with closed surveillance usually leads to good results.
